# Effectiveness of treatment of bedding and feces of laboratory animal with ozone

**DOI:** 10.1371/journal.pone.0266223

**Published:** 2022-04-06

**Authors:** Jiao-Jiao Qiao, Shan-Ni Wang, Jing-Jing Li, Li-Yu Chen, Mei-Mei Wang, Bin Yi, Qing-Xia Liu, Yun-Bo Liu, Chen Zhang, Paul Honess, Chang-Qing Gao

**Affiliations:** 1 Department of Clinical Laboratory, Xiang-Ya Hospital, Central South University, Changsha, China; 2 Center for the Study of Laboratory Animals, Xiang-Ya Hospital, Central South University, Changsha, China; 3 Department of Microbiology, Xiang-Ya School of Medicine, Central South University, Changsha, China; 4 Institute of Laboratory Animal Sciences, CAMS & PUMC, Beijing, China; 5 Department of Neurobiology, Beijing Key Laboratory of Neural Regeneration and Repair, School of Basic Medical Sciences, Capital Medical University, Beijing, China; 6 School of Veterinary Medicine and Science, University of Nottingham, Nottingham, United Kingdom; University of Alabama at Birmingham, UNITED STATES

## Abstract

**Background:**

The incineration and burying of the soiled bedding of laboratory animals, as well as using detergents to treat their feces, is hazardous to the environment. This highlights the need for an alternative, environmentally friendly solution for the treatment of the waste of laboratory animal facilities. This study aims to evaluate the efficacy of ozone disinfection of the soiled bedding and feces of laboratory animals.

**Methods:**

Two grams of soiled beddings were randomly sampled from the cages of mice and rats. These samples were mixed in a beaker with 40ml saline. Ozone was piped into the beaker at a concentration of 500mg/h. Samples were taken from the beaker at time 0min, 30min, 45min and 60min after ozone treatment for microbiological culturing in an incubator for 48h. Colony form unit of each plate (CFU/plate) at each time point were counted, the mean CFU/plate at each time point after ozone treatment were compared with that present at time zero. Feces of rabbits and dogs were treated and pathogens were counted the similar way as that of bedding of the mice and rats; samples being taken at 0min, 15min, 30min, 45min and 60min.

**Results:**

Pathogens were observed in beddings of both mice and rats as well as in feces of rabbits and dogs. Ozone treatment for 30min killed more than 93% of pathogens in the bedding of the two rodent species and 60min of treatment killed over 99% of pathogens. Treatment of rabbit and dog feces for 30min killed over 96% pathogens present, and 60min’s treatment killed nearly all the pathogens. Both Gram positive and Gram negative pathogens were sensitive to ozone treatment.

**Conclusion:**

Ozone treatment of bedding and feces is an effective and environment friendly way to deal with the waste of animal facilities, saving energy and potentially enabling their reuse as fertilizer.

## 1. Introduction

Cage bedding is one of the important factors that can influence the microenvironment of laboratory animals, and thus it may also impact on experimental results. Numerous studies have been conducted to improve the quality of bedding materials, but studies on how to treat soiled bedding before disposal are scarce [1–4]. The disposal of laboratory animal waste is an important issue as large amounts of soiled bedding, which contains not only excreta but potentially also tested compounds, from research and breeding facilities has potentially significant environmental impact. To date there seem to be no guidelines on with the safe and environmentally sensitive disposal of soiled bedding and other laboratory animal waste. Currently, in China, most soiled bedding is either incinerated or deep buried. Both methods are environment unfriendly.

Miyamoto et al. [5, 6] demonstrated that soiled bedding can be recycled for repeated use by applying soft hydrothermal processing. This processing not only enables reuse of the bedding, but actually improves the bedding’s capability to reduce in-cage levels of particulates and ammonia [6]. However, the process the authors describe is complicated, involving multiple machines and occupying considerable space, which is often very limited in animal facilities and this challenges the practical application of the proposed bedding treatment system.

Excreta of laboratory animals, including that of rabbits and dogs, are currently treated with chemical agents, such as those containing chlorine, which are typically hazardous to the environment. In short, the current treatment methods for animal waste, such as bedding and feces, are not optimal and need further investigation and better solutions.

Ozone has long been used to disinfect water and air, as well as other materials [7–11]. We therefore hypothesize that ozone treatment should be effective for inactivating pathogens in bedding and feces. Therefore, the aim of this study is to assess the effectiveness of ozone treatment for disinfecting soiled bedding and feces from laboratory animals, potentially enabling the use of this waste as fertilizer instead of its disposal via incineration or burying; thereby saving energy and reducing environmental impact.

## 2. Materials and methods

### 2.1 Bedding material sampling and treatment with ozone

Five mouse cages and five rat cages were randomly selected from a laboratory animal facility. Two grams of beddings were taken from each cage of the same species and mixed together in a plastic bag. Two grams of the combined bedding were placed in a beaker and mixed with 40ml of saline solution. Ozone was fed into the beaker with a pipe from an ozone generator (Model FL-8A, Feili Electric Appliance Technology Co., Ltd, Shen Zhen, China) at a concentration of 500mg/h. 20μL of solution samples were subsequently taken from the beaker at time 0min, 30min, 45min and 60min after ozone treatment and diluted by 1000 times. 5μL was taken from the diluted sample and added to blood agar plates (Columbia blood agar base medium, diameter 9 CM, Bioivd Biotechnology (Zhengzhou) Co., Ltd). The plates (five per time point) were incubated for 48h.

### 2.2 Animal feces sampling and treatment with ozone

Feces from eight rabbits, which were kept in the same laboratory animal facility, were collected in a beaker and mixed. One gram of the mixture was placed into a beaker and mixed with 60ml of saline. Ozone was fed into the beaker at a concentration of 500mg/h, the treatment continued for 60min with samples taken at intervals of 0min, 15min, 30min, 45min and 60min. Solutions taken at the 0min point were diluted 10 times with saline before sampling. The sampling at each time point was achieved by transferring 5μL of the treated solution onto a blood agar plate for culture. Each time point had five plates.

The treatment and sampling procedures for dog feces were the same as that used for rabbits.

### 2.3 Culture count and bacteria identification

After 48h of culturing, the bacterial colonies in each plate (colony form unit, CFU/Plate) were counted and the types of the bacteria were classified with matrix-assisted laser desorption/ionization time of flight mass spectrometry (MicroflexLT/SH. BRUKER, Germany).

### 2.4 Data analysis

Data of bacterial colony counts at different treatment time points were analyses using a related-samples Friedman two-way Analysis of Variance by Ranks (ANOVA) with post hoc pairwise comparisons (IBM SPSS Statistics v21). Proportions of Gram positive and Gram negative bacteria in samples before and after treatment were analysed using a 2x2 contingency table (Pearson Chi Square; IBM SPSS Statistics v21). Statistical significance for all tests was set at p<0.05.

## 3. Results

### 3.1 The disinfection efficacy of ozone on soiled beddings

Analysis shows that pathogen counts in samples taken from rat and mouse bedding at different time points during treatment with ozone are significantly different across all time points for each species (Friedman ANOVA: rat: F_3, 30_ = 74.95, p = 0.000; mouse: F_3, 15_ = 43.37, p = 0.000).

In rat bedding samples, post hoc pairwise comparisons against the untreated sample (time = 0min) reveal that ozone treatment to 30mins provides a significant reduction in bacterial colonies (F = 1.5, p = 0.000). Further treatment time does not produce significant reductions compared to preceding sampling time-points. However, treatment to 30, 45 and finally 60mins does provide additional benefits as reflected in greater effect size and an increasingly small rank mean and average pathogen count (see Table 1).

**Table 1:** The effectiveness of ozone treatment on soiled bedding of rats and mice.

Species	Treatment duration (min)	Average CFU/plate (n = 5)	Average logarithm	Percentage killed (%)
Rat	0	162.73		
	30	1.07	2.18	99.34%*
	45	0.20	2.91	99.88%*
	60	0.13	3.09	99.92%*
Mouse	0	124.80		
	30	8.33	1.18	93.32%*
	45	1.60	1.89	98.72%*
	60	0.33	2.57	99.73%*

*: Difference is significant when compared with the value at 0 min, P<0.05.

Data for mouse bedding treatment effect differ from those of the rat. In the mouse data only after 45min of treatment are bacterial colony counts significantly reduced compared to untreated samples (time = 0min) (F = 2.17, p = 0.000; see Table 1). As with rat bedding, treatment to 60min does result in the largest effect size (F = 2.833, p = 0.000) and also results in the lowest mean rank and average pathogen count of all sampling points.

As shown in Table 1, the ozone treatment of rat and mouse bedding for 30min killed 99% and 93% of pathogens in the beddings of the two species, respectively. Treatment for 60min killed over 99.70% of pathogens in both rat and mouse bedding.

### 3.2 The disinfection efficacy of ozone on animal feces

Analysis shows that pathogen counts in samples taken from feces of dogs and rabbits at different time points during treatment with ozone are significantly different across all time points (Friedman ANOVA: dog: F_3, 15_ = 58.61, p = 0.000; rabbit: F_3, 15_ = 55.36, p = 0.000).

In dog fecal samples, post hoc pairwise comparisons against the untreated sample (time = 0min) reveal that ozone treatment to 30min provides a significant reduction in bacterial colonies (F = 2.0, p = 0.005; Table 2). The effect size of the treatment continues to increase (also shown by progressive decrease in mean rank and average pathogen count) relative to time = 0min, up to time point t = 60mins (F = 6.697, p = 0.000; Table 2).

**Table 2:** The effectiveness of ozone treatment on the bacteria in dog and rabbit feces.

Species	Treatment duration	Average CFU/Plate (n = 5)	Average logarithm	Percentage killed
	(min)			(%)
Dog	0	4408.00		
	15	536.00	0.92	87.84%*
	30	143.60	1.49	96.74%*
	45	64.27	1.84	98.54%*
	60	36.33	2.08	99.18%*
Rabbit	0	936.00		
	15	155.47	0.78	83.39%*
	30	33.00	1.45	96.47%*
	45	1.60	2.77	99.83%*
	60	0.93	3.00	99.90%*

*: Difference is significant when compared with the value at 0 min, P<0.05.

Tests on rabbit feces data indicate that with ozone treatment a significant reduction in bacterial colony counts is only achieved at 30min (F = 3.811, p = 0.001; Table 2). The greatest effect of treatment is reached after 60min (F = 6.062, p = 0.000; Table 2).

As indicated in Table 2, ozone treatment of dogs and rabbits feces for 30min killed over 96% of pathogens for both species, and treatment for 60min killed nearly all of the pathogens in the feces of the both species (dog: 99.18%, rabbit: 99.9%).

### 3.3 Impact of ozone treatment on pathogen species in bedding

Table 3 shows the changes pathogen species profile in mouse and rat bedding before (time = 0min) and after (time = 60min) treatment with ozone. In rat bedding, before ozone treatment, there were six species of pathogen, of which five were Gram positive and one was Gram negative. After 60min of ozone treatment there was only one a Gram negative bacterium species (*P*. *pasteurii*) still present.

**Table 3:** The impact of ozone treatment on pathogens in the bedding of rats and mice.

Animal species	Pathogen	Gram staining	Before treatment	After treatment	Characteristics
Rats	*Aerococcus viridans* *	G+	+	-	Potential pathogen of bacteremia; can cause urinary tract infection and occasionally causes endocarditis [22].
	*Alcaligenes faecalis* *	G-	+	-	Potential pathogen; can lead to opportunistic infections in humans [23].
	*Bacillus gibsonii*	G+	+	-	
	*Paenalcaligenes hominis* *	G-	+	-	Can cause colitis, etc [24, 25].
	*Psychrobacter pasteurii*	G-	+	+	Potential pathogen
	*Vagococcus lutrae* *	G+	+	-	
Mice	*Aerococcus viridans* *	G+	+	-	Potential pathogen of bacteremia; can cause urinary tract infection and occasionally causes endocarditis [22].
	*Corynebacterium stationis*	G+	+	+	
	*Hydrogenophaga pseudoflava*	G-	+	-	
	*Jeotgalicoccus haiotolerans*	G+	+	-	
	*Myroides odoratimimus* *	G-	+	-	Potential pathogen; can lead to cellulitis and necrotizing fasciitis [26], pneumonia [27], endocarditis and urinary tract infection [26, 27]
	*Paenalcaligenes hominis* *	G-	+	+	Can cause colitis, etc [24, 25].
	*Psychrobacter pasteurii*	G-	+	+	
	*Staphylococcus sciuri* *	G+	+	-	Pathogenicity and drug resistance [23].

Notes: The bacteria listed above are the results of comparison with the Clinical Pathogenic Microorganism Database。

(*: pathogenic

+: present

-: absent).

In the bedding of mice, there were eight species of pathogens (Gram positive = 4, Gram negative = 4) present before ozone treatment and only three bacteria species (Gram positive = 1, Gram negative = 2) persisted after treatment for 60min.

### 3.4 Impact of ozone treatment on pathogen species in feces

Table 4 illustrates the changes in pathogen species profile of the feces of rabbits and dogs before (time = 0min) and after (time = 60min) ozone treatment. In rabbit feces, there were two species of pathogens present before ozone treatment (both Gram positive) and only one of these was present after treatment. In dog feces, there were three bacterial species present before treatment (Gram positive = 2, Gram negative = 1) and two of these were still present after ozone treatment (Gram positive = 1, Gram negative = 1).

**Table 4:** The impact of ozone treatment on pathogens in the feces of rabbits and dogs.

Animal species	Pathogens	Gram staining	Before treatment	After treatment	Characteristics
Rabbit	*Bacillus pumilus* *	G+	+	-	Sepsis and immune impairment in newborns [28, 29], central venous catheter infection [30], and skin infection [31].
	*Enterococcus hirae*	G+	+	+	
Dog	*Bacillus pumilus* *	G+	+	-	Sepsis and immune impairment in newborns [28, 29], central venous catheter infection [30], and skin infection [31].
	*Escherichia coli* *	G-	+	+	Causes diarrhea and parenteral diseases [32].
	*Enterococcus faecium* *	G+	+	+	Pathogenic [33].

Notes:The bacteria listed above were identified via comparison with the Clinical Pathogenic Microorganism Database。(*: pathogenic

+: present

-: absent).

### 3.5 Sensitivity of bacteria to ozone

Table 5 shows that before ozone treatment, there were eight Gram negative pathogens and 11 Gram positive pathogens in the bedding of rats and mice and the feces of dogs and rabbits. After ozone treatment there were four Gram negative and three Gram positive pathogens present. There is no significant difference in sensitivity to ozone treatment between Gram negative and positive pathogens (χ^2^_1,26_ = 0.465, p = 0.665).

**Table 5:** Comparison of sensitivity of Gram^+^ and Gram^-^ bacteria to ozone treatment of the waste of rats and mice (bedding), and rabbits and dogs (feces).

	Before ozone treatment	After ozone treatment
Gram^+^	11	3
Gram^-^	8	4

Note: Before ozone treatment, there were 8 gram staining negative pathogens and 11 gram staining positive pathogens in the bedding of rats and mice as well as in the feces of dogs and rabbits, after ozone treatment there were 4 gram staining negative and 3 gram staining positive pathogens present. There is no significant difference in the sensitivity to the treatment of ozone between gram staining negative and positive pathogens.

## 4. Discussion

In this study we found that there are pathogens in the soiled beddings of rat and mice and in the feces of dogs and rabbits. Ozone treatment can effectively kill these pathogens, and both Gram positive and negative pathogens are sensitive to this treatment.

The materials used as bedding have been found to hold a variety of bacteria, even when new [12], therefore they are now autoclaved before being used. Our current study confirms that various pathogens are present in the soiled bedding of specific pathogen free (SPF) as well as non-SPF facilities [13–19].

Domestic dogs may carry a range of pathogens [20, 21]. In dogs used in research, zoonotic and other pathogens, such as *Salmonella spp*., pathogenic dermal fungi, *Brucella spp*. and Rabies Virus that may cause serious illness and confound research, could be present and should be excluded according to the National Standards of China [20]. However, some pathogens that have only minor effects may still be present. In the current study, we found some pathogens that are not on the health-screening list of the National Standards. The situation is similar for rabbits.

The treatment of waste, such as bedding and feces, from laboratory animal facilities remains a significant challenge internationally [5, 6]. Incinerating and burying waste uses energy and contaminates the environment. A previously proposed and tested system for recycling bedding is impractical in most facilities due to space requirements for additional machinery [5, 6]. Using hydrogen peroxide vapour can inactivate pathogens in bedding, but this disinfection only works well against pathogens on the surface of waste. The current study demonstrates that treatment with ozone can effectively inactivate nearly all pathogens in the beddings, whether on the surface or in the core of the waste.

In China, the feces of research animals, including that of dogs and rabbits, is currently treated with agents containing chloride. Our study demonstrates that ozone treatment can effectively inactivate nearly all the pathogens in the feces of these animals. Unlike chloride agents which require special removal and treatment, ozone automatically decomposes in between 30 minutes and four hours, and thus is much less damaging to the environment.

As the ozone we used is generated from the air, it is nearly costless and it is certainly affordable. When using ozone to disinfect the beddings, a container is always used and the ozone is constricted in the container, thus will not contaminate the air.

Taken together, the ozone treatment of bedding and feces is an effective and environmentally friendly way to deal with the waste of animal facilities; saving energy and potentially enabling the reuse of waste as a fertilizer.
